# Gamma Synchronization Influences Map Formation Time in a Topological Model of Spatial Learning

**DOI:** 10.1371/journal.pcbi.1005114

**Published:** 2016-09-16

**Authors:** Edward Basso, Mamiko Arai, Yuri Dabaghian

**Affiliations:** 1 Department of Physics, Rice University, Houston, Texas, United States of America; 2 Department of Mathematics, Tokyo Women’s Christian University, 2-6-1 Zempukuji, Suginami-ku, Tokyo, Japan; 3 Jan and Dan Duncan Neurological Research Institute, Baylor College of Medicine, Houston, Texas, United States of America; 4 Department of Computational and Applied Mathematics, Rice University, Houston, Texas, United States of America; University of Pittsburgh, UNITED STATES

## Abstract

The mammalian hippocampus plays a crucial role in producing a cognitive map of space—an internalized representation of the animal’s environment. We have previously shown that it is possible to model this map formation using a topological framework, in which information about the environment is transmitted through the temporal organization of neuronal spiking activity, particularly those occasions in which the firing of different place cells overlaps. In this paper, we discuss how gamma rhythm, one of the main components of the extracellular electrical field potential affects the efficiency of place cell map formation. Using methods of algebraic topology and the maximal entropy principle, we demonstrate that gamma modulation synchronizes the spiking of dynamical cell assemblies, which enables learning a spatial map at faster timescales.

## Author Summary

One of the goals of theoretical systems neuroscience is to connect parameters of neuronal activity observed in electrophysiological experiments, such as cell firing rates, frequencies and amplitudes of the brain rhythms, with cognitive phenomena that emerge at the scale of large groups of cells. In previous work, we proposed an approach for modeling one such phenomenon: spatial learning in the mammalian hippocampus. This approach rested on the hypothesis that hippocampal neurons encode a rough-and-ready topological map of an environment, whereas geometric details likely come from multiple inputs from other brain regions. A key property of this model is that it allows us to estimate the effect produced by different parameters of neuronal activity on spatial learning. In particular, it we showed that theta oscillations strongly enhance the ability of the place cell ensembles to learn topologically accurate spatial maps. In this work, we show that synchronization of neuronal spiking activity by the other major component of the oscillating extracellular cell potential—the gamma rhythm—also enhances both spatial learning and the retrieval of spatial memories at the physiological timescale.

## Introduction

The mammalian hippocampus plays a key role in spatial cognition. Hippocampal place cells manifest remarkable spatial specificity of spiking activity: they fire only in select locations in the environment, known as place fields [1]. As a result, place cell spike trains contain information about the animal’s current location [2], as well as its future [3] and past [4] navigation routes, both in the wakeful state and even in sleep [5]. Moreover, damage to the hippocampal network impairs spatial learning and navigation planning [6, 7]. It is thus believed that the population of place cells encodes a cognitive map of the environment that serves as the basis of animal’s spatial awareness [8, 9].

### Topology vs. geometry

For years it has been assumed that the cognitive map is Cartesian, containing detailed information about locations, distances and angles. Undoubtedly, such information is provided by various brain regions, but increasing evidence suggests that the hippocampal map is topological in nature. For example, electrophysiological recordings in morphing environments demonstrate that the spatial order (overlaps, adjacency and containment) is preserved, even in the face of deformations of the environment that cause the place fields to stretch or change shape [10–14]. In other words, the sequential order of place cell activity induced by the animal’s moves through morphing environment remains invariant, at least within a certain range of geometric transformations [15–17]. This implies that place cell spiking encodes a rough-and-ready framework into which other brain regions integrate more detailed metrical information [17–21].

What sorts of neuronal computations could produce such a framework [22–27]? The approach proposed in [28–30] exploits the connection between the place field map and the Alexandrov-Čech theorem, according to which the pattern of overlaps between regular spatial regions *U*_1_, *U*_2_, …*U*_*N*_ covering a space *X* encodes the topological structure of *X* [32]. The construction suggested by the Alexandrov-Čech theorem is the following. If the regions *U*_*i*_ are represented as vertices, pairs of overlapping regions *U*_*i*_ ∩ *U*_*j*_ ≠ ∅, as links between these vertices, the triples *U*_*i*_ ∩ *U*_*j*_ ∩ *U*_*k*_ ≠ ∅ as the facets between these links and so forth, then the resulting simplicial complex N is topologically equivalent to *X* (see Glossary in the Methods section and S1 Fig).

The fact that the place fields produce a dense cover of the environment suggests that the pattern of overlaps between them contains the information required to represent the environment’s topology, which we propose holds the key to the way in which the hippocampus encodes its topological map of a given space. Note that the domains where several place fields overlap are precisely the ones where the corresponding place cells cofire: the information about the overlap of place fields is represented via place cell coactivity, which suggests that the Alexandrov-Čech construction can be carried out not only via the geometric pattern of the place field overlaps, but also through analysis of place cell coactivities.

### Topological model

**Fig 1:**
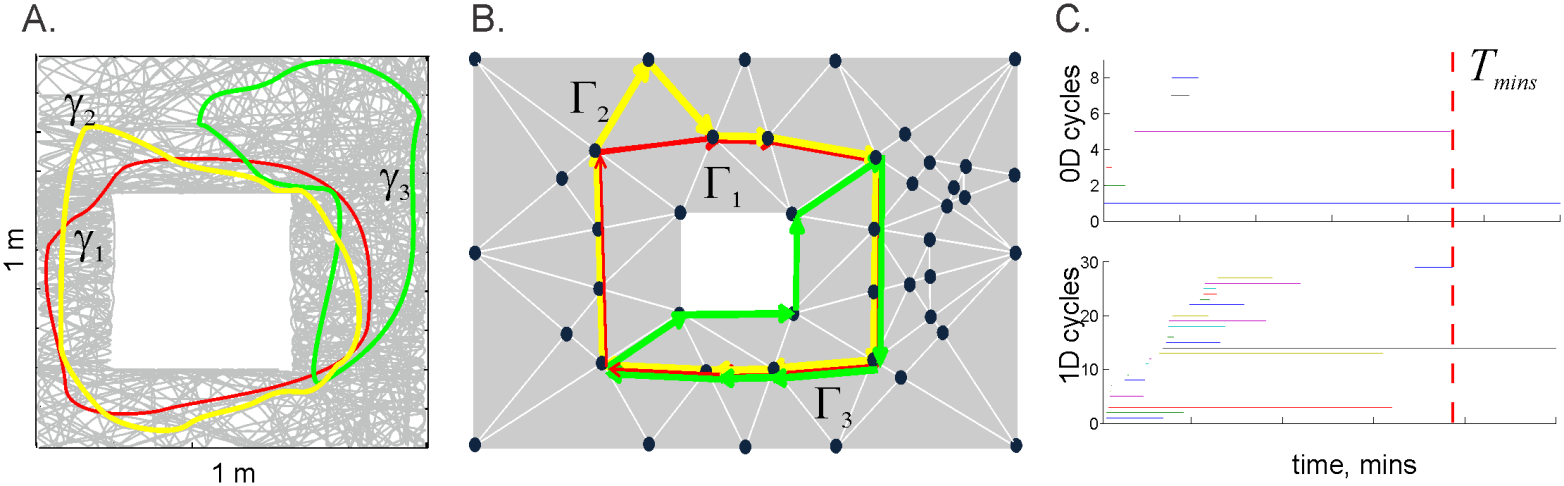
An animal’s movements through a given space are represented in the coactivity complex. A: Two topologically equivalent paths in a physical environment, *γ*_1_ and *γ*_2_ (this commonly used notation for the paths is unrelated to *γ*-rhythm), encircle an obstacle (white square) and are therefore non-contractible. The path *γ*_3_ does not encircle the obstacle and therefore is contractible and topologically inequivalent to *γ*_1_ and *γ*_2_. B: A schematic representation of the 2*D* skeleton of the coactivity complex $T_{\sigma }$ (vertices shown as black dots, the 1*D* links as white lines and 2*D* facets as grey triangles) and of the simplicial paths Γ_1_, Γ_2_ and Γ_3_, which represent the physical paths *γ*_1_, *γ*_2_ and *γ*_3_. The topological equivalences and inequivalences between the simplicial paths (Γ_1_ ≅ Γ_2_ and Γ_1_ ≇ Γ_3_, Γ_2_ ≇ Γ_3_) provide qualitative information about the physical paths, encoded via place cell coactivity. Since we are concerned primarily with representing the topological properties of the navigational paths, in the following we discuss only the 2*D* skeleton of the coactivity complex. C: Timelines of the topological loops encoded in the coactivity complex. As the animal begins to explore its environment, the coactivity complex contains many spurious topological loops (gaps in the information about that part of the environment) most of which do not represent the physical obstacle. This “topological noise” in the depicted graph disappears after about five minutes, which marks the learning time, *T*_min_—the moment when the correct topology of space has emerged. One 1*D* loop represents the obstacle and one 0*D* loop informs us that the environment is connected.

The details of the topological model of the hippocampal map are discussed in [29, 30]. In brief, the idea is to represent the combinations of coactive place cells (*c*_1_, *c*_2_, …, *c*_*p*_) as coactivity simplexes, *σ* = [*c*_1_, *c*_2_, …, *c*_*p*_]—combinatorial representations of multi-dimensional polyhedra (see Methods). Together, these coactivity simplexes form a simplicial coactivity complex $T_{\sigma }$. In this construction, the individual cell assemblies (i.e., a group of neurons that jointly drive a downstream readout neuron), provide local information about a given space; joined together into a neuronal ensemble (i.e., a population of cell assemblies), they represent the space as whole. By analogy, a collection of individual simplexes representing connected locations, together form a simplicial complex which represents environment as a whole. Numerical simulations demonstrate that $T_{\sigma }$ captures the topological structure of the environment and serves as a schematic representation of the hippocampal map [29, 31]. For example, the sequences of place cell combinations ignited along the paths traversed by the animal are represented in $T_{\sigma }$ by chains of coactivity simplexes—the simplicial paths [33, 34]. A non-contractible simplicial path may represent a navigational path that encircles a physical obstacle, whereas topologically trivial simplicial paths correspond to contractible routes in the physical space (Fig 1A and 1B).

The complex $T_{\sigma }$ begins to form as soon as the rat starts navigating. Every detected instance of place cell coactivity contributes a simplex to $T_{\sigma }$. At the early stages of navigation, when only a few cells have time to produce spikes, the coactivity complex is small, fragmented, and contains many gaps (in topological terms, “holes”), most of which do not represent physical obstacles in the environment. Such holes tend to disappear as spatial learning continues. Therefore, the minimal time, *T*_min_, after which the topology of $T_{\sigma }$ matches the topology of the environment, or more precisely, when the correct number of topological loops emerges, can be viewed as a theoretical estimate of the time required to learn the hippocampal map (Fig 1C, [29, 30]).

### Physiological parameters

An important property of the model is that the structure of the coactivity complex $T_{\sigma }$ and the time course of its formation during learning are sensitive to various parameters of the neuronal firing statistics, which allows us to study the effect that changes in any of these parameters (e.g., firing rate, place field size, number of neurons) produce in the ability of the ensemble to correctly learn a space. For example, if the firing rate slows, the system can compensate with a change to the place field size or the number of neurons in the ensemble, but only up to a point: beyond certain limits, the assembly will not be able to learn efficiently, or even at all [29]. As another example, the oscillations of the extracellular electrical field potential, typically referred to as the local field potential (LFP), are known to modulate place cells activity at several timescales: each place cell tends to spike within a small range of the phases of the theta component of the LFP (*θ*, 4–12 Hz [35]), which depends on the distance that the animal has traveled into the corresponding place field. As a rat moves through the place field, the preferred *θ*-range of a place cell progressively decreases with each new *θ*-cycle, a phenomenon known as *θ*-phase precession [36]. The preferred *θ*-phases of different cells are additionally synchronized by the second major component of the LFP, the gamma rhythm (*γ*, 30–80 Hz, [37]). In fact, the period of the more rapid *γ*-rhythm, *T*_*γ*_, is believed to define the range of the preferred phases within the slower *θ*-rhythm; on average one *θ*-period, *T*_*θ*_, contains about seven *γ*-cycles, *T*_*θ*_ ≈ 7*T*_*γ*_ (see [38] and S2A Fig).

Numerous experimental [39–43] and theoretical [38, 44–47] studies demonstrate that both *θ*- and *γ*-waves play key roles in spatial, working, and episodic memory functions. Most theoretical analyses have addressed the way in which the *γ*-synchronization affects the informational contents of spiking in small networks or in individual cells, but the topological approach allows us to model the formation of cognitive map as a whole. For example, it was used in [30] to demonstrate that *θ*-precession makes otherwise poorly-performing ensembles more efficient at spatial learning.

The present analysis applies the topological model to study the effect of *γ*-waves on spatial learning and to demonstrate that *γ*-synchronization of place cell spiking activity enables the encoding or retrieval of large-scale spatial representations of the environment by integrating place cell coactivity at a synaptic timescale.

### Brain rhythms in the topological model

Computational modeling of *θ*-phase precession is relatively straightforward. At a basic level, it amounts to imposing a particular relationship between a place cell’s spiking probability, the phase of the *θ*-wave and the distance that the animal has traveled into the corresponding place field [48] (see Methods). The effects of the *γ*-rhythm are, however, more diverse. Electrophysiological experiments suggest that there exist at least two types of place cells: “TroPyr” cells that spike at the trough of the fast *γ*-wave (50–80 Hz) and “RisPyr” cells that fire at the rising phase of slow *γ*-waves, overriding *θ*-precession [49–51]. Although we can model both S2 Fig with our approach (see Methods), in the following we will model only the TroPyr cells that exhibit more robust firing patterns and higher firing rates, and therefore may play a primary role in producing the cognitive map [29, 30].

#### *γ*-modulation of spiking

Physiologically, the *γ*-wave represents fast oscillations of the inhibitory postsynaptic potentials. As the amplitude of *γ* drops at a certain location, the surrounding cells with high membrane potential spike [53–55]. As a result, the preferred *θ*-phase of several cells becomes synchronized with a *γ*-trough, which thereby gates the place cell coactivity. The literature refers to such groups of coactive place cells as “dynamical cell assemblies” (see [60–62] and S2A Fig).

Modeling *γ*-modulation therefore requires adjusting the times of the *θ*-modulated spikes closer to the troughs of the *γ*-wave [57]. Algorithmically, this task is similar to the task of distributing particles stochastically over the wells of a 1*D* potential energy field, which is solved based on the Maximum Entropy Principle [58]. The probability that a particle lands at point *x* in a potential *U*(*x*) is *p* ∼ *e*^−*βU*(*x*)^, where the parameter *β* controls the spread of locations around the minima of *U*(*x*). The lower the *β*, the more random the distribution. In this work we will borrow terminology from statistical physics, where the parameter *β* is the reciprocal temperature of the system, and lower values of *β* mean warmer temperatures (higher stochasticity) and higher values of *β* mean cooler temperatures (lower stochasticity; see S2B Fig).

**Fig 2:**
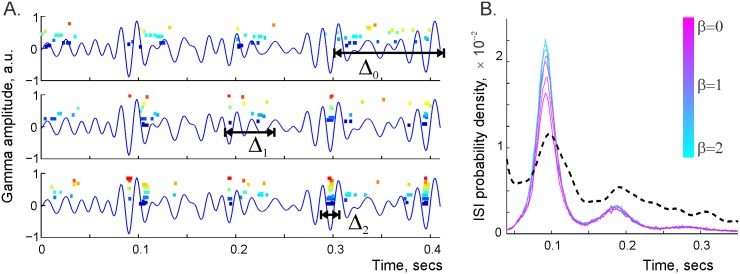
Gamma synchronization. A: Without coupling with the *γ*-wave (*β* = 0, top panel) the simulated place cell spikes are scattered diffusely over the time axis. The temporal spread of the place cell coactivity exceeds a *θ*-period, Δ_0_ ≈ 1.5*T*_*θ*_, *T*_*θ*_ ≈ 125 msec. At *β* = 1, the intervals of place cell coactivity concentrate near domains of high *γ*-amplitude, Δ_1_ ≈ 0.5*T*_*θ*_. At *β* = 2, the spikes accumulate near the *γ*-troughs, Δ_2_ ≈ *T*_*γ*_, thus producing dynamical cell assemblies (bottom panel). B: The statistics of interspike intervals (ISI) for different *β*s. The black dashed line shows the distribution of the time intervals between deep *γ*-troughs (deeper than two standard deviations of *A*_*γ*_(*t*) from the mean). As *β* increases, the intervals between spikes are more controlled by the deep troughs (where the amplitude exceeds three standard deviations of *A*_*γ*_(*t*) above the mean). Note that the tendency for spikes produced by the same place cell to appear within the same *γ*-cycle can be viewed as a basic model of bursting [52].

Viewing the *γ*-amplitude, *A*_*γ*_(*t*), as an inhibitory potential extended over the time axis, we confined the place cell firing to the troughs of the *γ*-wave by modulating their firing rates with the factor $e^{-\beta_{\gamma }A_{\gamma }(t)}$. Thus, the parameter *β*_*γ*_ controls the temporal spread Δ_*β*_ of spikes produced by the dynamical cell assemblies. For small *β*_*γ*_, the cell assemblies are “hot,” meaning their spikes are spread diffusely near the *γ*-troughs. For large *β*_*γ*_ the assemblies are “cold,” their spikes “freeze” at the *γ*-troughs (Fig 2 and S3 Fig). In particular, the case in which the spike trains are uncorrelated with the *γ*-troughs corresponds to the limiting case of an “infinitely hot” (*β*_*γ*_ = 0) hippocampus, modeled in [30].

To our knowledge, the statistics of the temporal spreads of the spikes produced by dynamical cell assemblies have not been studied, but the neurophysiological literature suggests that a typical spread is about one *γ*-period (Δ ≈ *T*_*γ*_ ≈ 20 msec) [59–62]. This implies that the effective temperature of these hippocampal cell assemblies is comparable to the mean *γ*-trough amplitude $1/\beta_{\gamma }\approx {\bar{A}}_{tr}$ (see Methods). In the following discussion, it will be convenient to scale the amplitude of the *γ*-wave according to its standard deviation from the mean, *σ*_*γ*_, *A*_*γ*_(*t*) → *A*(*t*) = *A*_*γ*_(*t*)/*σ*_*γ*_. In turn, this entails the corresponding scaling of the inverse temperature, yielding a parameter *β* = *β*_*γ*_ *σ*_*γ*_ with the “physiological” range between 0.5 ≲ *β* ≲ 2.

#### Reading out place cell coactivity

The spiking signals produced by the place cells are transmitted to a population of neurons downstream from the hippocampus. In the reader-centric approach to information processing in the hippocampal network [31, 61], the cell assemblies are viewed not simply as occasional combinations of coactive place cells, but as functionally interconnected cell groups that exhibit repeated coactivity and jointly trigger responses from their respective readout neurons. In turn, the readout neuron *n*_*σ*_ spikes upon receiving a sufficient amount of timed EPSP inputs over a certain period *w*_*σ*_, defined as the “integration window” [63–65], which is the only parameter describing readout neurons in the following discussion. Clearly, different readout neurons may integrate inputs over different time intervals. However, in order to simplify the approach, we will describe the entire population of the readout neurons using a single parameter *w*_*σ*_ = *w*, viewed as the average time over which a typical readout neuron accumulates EPSP inputs [30].

#### *θ* and *γ* synchronicity

In our previous study [30], we modeled assemblies of independently *θ*-precessing place cells simply as groups of neurons that happened to produce spikes within a certain *w*-period. The model predicted that the correct spatial information is reliably encoded if the coactivity inputs are identified over the *θ*-timescale (*T*_*θ*_ ≲ *w* ≲ 2*T*_*θ*_). However, as *w* shrinks, the chance of producing and detecting the coactivities within a *w*-period diminishes, and the topological map takes longer to form. For the intermediate range of values (3*T*_*γ*_ ≲ *w* ≲ 3*T*_*θ*_), the learning time is approximately inversely proportional to *w*, but as *w* reduces to the *γ*-period, the pool of detected place cell coactivities more frequently fails to capture the topological structure of the environment or requires a much longer time to produce it, exhibiting high variability of *T*_min_ upon *w*. In contrast with these results, experimental studies have shown that the synchronicity of the place cell assemblies is best manifested precisely at the *γ*-timescale [60–62]. This implies that the hippocampal network is capable of producing large-scale spatial maps based on *γ*-timescale readouts. From this, we hypothesized that the failure of the previous (*θ*-driven) topological model to capture this empirical evidence might be due to poorer synchronization of independent neurons driven by a common *θ*-pacemaker, rather than to the physiological cell assemblies that are additionally synchronized through synaptic interactions [66].

In the present analysis, we use the effective temperature 1/*β* to describe phenomenologically these additional synchronization mechanisms. As illustrated in Fig 2, the parameter *β* controls the temporal spread of spiking activity in cell assemblies Δ_*β*_ independently from *w* and allows us to transition from modeling desynchronized cell assemblies to modeling cell assemblies that are tightly coupled with *γ*-troughs. The results shown in Fig 2 suggest also that binding the coactivity of place cell assemblies within *γ*-periods (Δ_2_ ≈ *T*_*γ*_) should significantly reduce the time required by the downstream networks to detect place cell coactivity. Thus, *γ*-synchronization may enable us to construct a reliable neuronal representation of space within a much tighter temporal window, *w* ≈ *T*_*γ*_, which provides a possible explanation for its functional importance.

## Results

**Fig 3:**
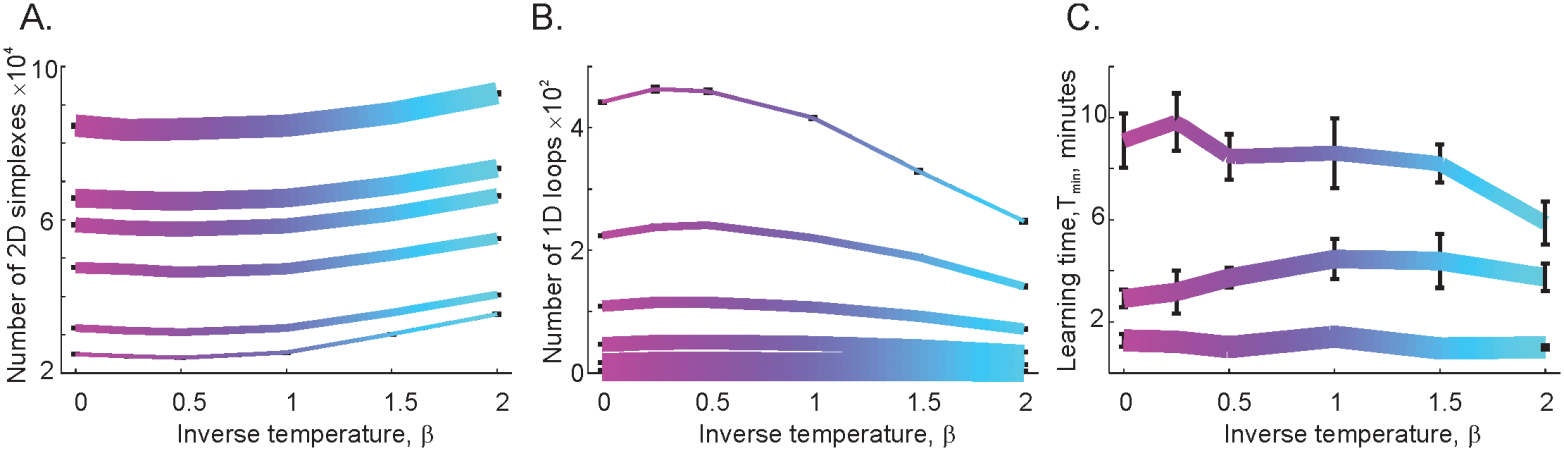
Influence of *γ*-modulation on spatial learning in a cell assembly network with coincidence detector readout neurons. There are two major parameters of the model: the mean width of the temporal window *w* over which the postsynaptic readout neurons integrate spikes from the place cell assemblies (*w*_1_ = 2*T*_*θ*_, *w*_2_ = 1.2*T*_*θ*_, *w*_3_ = 0.8*T*_*θ*_, *w*_4_ = 0.5*T*_*θ*_, *w*_5_ = 0.3*T*_*θ*_, *w*_6_ = 0.2*T*_*θ*_, with larger windows *w* represented by thicker lines), and the effective temperature 1/*β* which controls the clustering of place cells’ spikes around the troughs of the *γ*-wave (Fig 2). Larger values of *β* (indicated by the blue color of the colormap) correspond to tighter coupling between the place cell’s spiking probability and the *γ*-amplitude (S2 Fig). A: The number of 2*D* simplexes, *N*_2_, in the coactivity complex $T_{\sigma }$ as a function of *β*, for different *w*’s. For large integration windows (thicker lines), coupling with the *γ*-wave produces no significant effect: the *N*_2_ changes less along the *β*-axis. As *w* decreases, the number of simplexes drops (the thinner lines lay below the thicker ones). However, the smaller the integration window *w*, the greater the number of coactive place cell combinations, produced by the cooling of the cell assemblies. For *w* = *T*_*γ*_ (bottom curve) the number of 2*D* simplexes grows by 40% as *β* increases from 0 to 2. B: Shrinking the integration window *w* increases the total number of topological loops observed in $T_{\sigma }$ during the course of learning, whereas cooling down the coactivity complex reduces this number. For example, the number of spurious loops in cold coactivity complexes (cold loops, *β* = 2) at *w* = *T*_*γ*_ is half the number of those in hot complexes (hot loops, *β* = 0). C: The learning time *T*_min_ grows as *w* shrinks and tends to decrease as a function of *β*. However, note that even cold simplicial complexes fail to produce the correct topological maps for small *w*. In the particular map illustrated here (mean place field size *s* = 24 cm, mean firing rate *f* = 20 Hz, *N*_*c*_ = 450 cells), learning time diverges at *w* ≥ 0.5*T*_*θ*_.

To assess the effects of *γ*-waves on the ability of place cells to encode spatial information, we built the coactivity complex using *γ*-modulated spike trains for different *β*s and studied its topological properties for a set of *w*’s, including the values for which the independent *θ*-precessing place cells fail to produce correct topological maps. The results shown in Fig 3 demonstrate that, at large integration windows (*w* ≥ *T*_*θ*_, fat lines), tightening the cell assemblies around the *γ*-troughs does not produce a significant effect on either the structure of $T_{\sigma }$ or on the times required to learn the map *T*_min_. This outcome is easy to explain: if the readout neurons accumulate EPSPs at the *θ*-timescale, i.e., over hundreds of milliseconds, the temporal arrangement of the spikes at the *γ*-timescale does not change the combinations of coactive place cell detected downstream. In other words, no matter how the *γ*-tuned spikes are spread inside a *θ*-wide window *w*, the coactivity simplexes, and thus the coactivity complex, remain the same, yielding the same topological information after the same learning period. As *w* decreases, the temporal spread of the poorly synchronized, hot place cell assemblies (*β* < 1) begins to exceed *w*. As a result, only a fraction of the coactive cells can be detected downstream, which leads to a decrease of the number of simplexes in $T_{\sigma }$ and to a proliferation of spurious topological loops during the learning period. Moreover, many of these loops persist indefinitely, preventing the appearance of the correct topological information even at the intermediate values of *w* (Fig 3C).

In contrast, the behavior of the cold cell assemblies (*β* > 1, the blue ends of the graphs) is different. First, the number of 2*D* simplexes increases, because the size of the cell assemblies increases with increasing *β* (Fig 3A). Second, colder coactivity complexes $T_{\sigma }$ yield fewer, faster-contracting spurious loops (Fig 3B and S4 Fig). Third, the learning times drop significantly: for *β* = 2, the *T*_min_ computed for *w* = 0.5*T*_*θ*_ is reduced by about 50% compared to the desynchronized, *β* = 0 case, which indicates that *γ*-synchronization promotes the formation of a topological map based on the coactivity information transmitted to the downstream networks at times shorter than one *γ*-cycle (Fig 3C and S5 Fig).

Nevertheless, the results shown in Fig 3 typically do not extend to the *γ*-timescale of *w*. The inputs collected from the cell assemblies which cooled to the physiological range of *β*s (0.5 ≲ *β* ≲ 2) at *w* < 0.3*T*_*θ*_ often failed to produce an accurate map of the environment. This suggests that producing a correct neuronal map of space within a biologically plausible learning time using *w* ≈ *T*_*γ*_ requires further cooling of $T_{\sigma }$ (by increasing *β* indefinitely, the cell assemblies can be made to fire as tightly at the troughs as desired). Thus, in order to keep the parameter *β* within the physiological range, we have deployed an alternative approach.

### Clique coactivity complexes

In the above discussion, the central construction of the model, which is the coactivity complex $T_{\sigma }$, was introduced as a schematic representation of the place field map [31]. However, as shown in [67, 68], a coactivity complex can be built not only by detecting higher-order cofiring events that directly mark the locations where several place field overlap, but also by integrating the information provided by the lower-order place cell coactivity. Physiologically, the latter option corresponds to the cell assembly network in which the readout neurons integrate lower-order coactivity inputs over a working or intermediate memory timescale, rather than merely react to cofiring as all-or-none coactivity detectors [69, 70].

To model a network of cell assemblies driving a population of input-integrator readout neurons, we used the following approach. First, we detected the lowest-order, pairwise place cell coactivities and used them to build a connectivity graph *G* (see [67] and S6 Fig). Fully interconnected subgraphs of *G* are called cliques (see Methods); cliques of *G* are identified with the simplexes of a new *clique coactivity complex* $T_{\varsigma }$. A key property of this algorithm is that the connections constituting a clique or a simplex do not have to be detected simultaneously but can be accumulated over an extended period of time. For physiological accuracy, we restrict this period to 10 mins or less, which results in a coactivity complex whose simplexes emerge over working or intermediate memory intervals.

Although the algorithms for constructing temporal simplicial and clique complexes seem quite different, the actual difference between these two coactivity complexes is not as large. First, as shown in [31, 67], most simplexes of $T_{\varsigma }$ correspond to the simplexes of $T_{\sigma }$ and vice- versa: the identities of the cell assemblies are largely the same, only the time course of their construction changes. Furthermore, the topological structures of these complexes are quite close. Second, most pairwise connections within the cliques of *G* are produced almost simultaneously while the rat traverses the region where several place fields overlap. In other words, most cliques appear at once, just as the simplexes do, and only a relatively small number of the maximal cliques are actually “corrected” over time [68]. Nevertheless, this effect does improve the overall performance of the clique coactivity complexes, which typically produce far fewer spurious topological loops and shorter learning times *T*_min_ than those produced by simplicial coactivity complexes.

**Fig 4:**
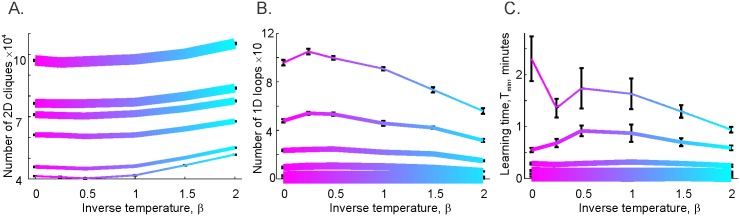
Influence of the *γ*-modulation on spatial learning in cell assembly network with input integrator readout neurons: clique complexes. A: The dependence of the number of triple connections in the clique coactivity complex $T_{\varsigma }$ is similar to the dependence of the number of 2*D* simplexes in the simplicial coactivity complex. As the integration window narrows (same range of *w*’s as on Fig 3), the number of triple connection cliques drops. Cooling down the assemblies produces no significant effect at large integration windows, but does increase the number of triple connections for small *w*’s (by about 25% for *w* ≈ *T*_*γ*_). B: The total number of topological loops observed in the clique coactivity complex $T_{\varsigma }$ is reduced with cooling for small *w*’s, in a way similar to the case of the coincidence detection (Čech) coactivity complex. At the *γ*-timescale, *w* ≈ *T*_*γ*_, the tendency of the shrinking *w*’s to cause the clique complex to form large numbers of topological loops is nearly compensated by cooling down $T_{\varsigma }$: the number of cold loops (*β* = 2) in $T_{\varsigma }$ is about 50% of the number hot loops (*β* = 0). Note that despite similar qualitative behaviors, the scales of $N_{l_{1}}^{\sigma }$, and $N_{l_{1}}^{\varsigma }$ are different: the clique complex produces fewer spurious loops than the simplicial complex. C: Learning times grow as a function of *w*; however, for the clique complex they remain finite even for *w* ≈ *T*_*γ*_. The more tightly clustered the clique complex is around the *γ* troughs, the more rapidly it learns, especially for small *w*.

Implementing the *γ*-synchronization mechanism in an integrator model yields the results illustrated in Fig 4. First, the structure of the graphs on Figs 3A and 4A is qualitatively similar, though the pool of third-order cliques is slightly larger than the pool of 2*D* simplexes. This is because not every clique makes a simultaneous appearance as a simplex, but every simplex can be viewed as an instantly detected clique. The behaviors of the topological loops in $T_{\varsigma }$ and in $T_{\sigma }$, shown in Figs 3B and 4B are similar as well. The *γ*-synchronization reduces the number of cold, spurious loops in both types of complexes (S7 Fig). Physiologically, this implies that a *γ*-rhythm produces the same organizing effect on the activity of cell assembly network, whether the latter is based on a coincidence detector or on the input integrator readout neurons. However, it should be noted that, for all *β*s, the number of loops in $T_{\varsigma }$ is smaller than in $T_{\sigma }$ by an order of magnitude, illustrating the efficiency of the input integrating readout neurons. Most importantly, the integrator complex $T_{\varsigma }$ produces finite learning times at the *γ*-timescale integration window, *w* ≈ *T*_*γ*_. This demonstrates that the hippocampal network can produce a spatial map by reading out *γ*-synchronized place cell coactivity at the *γ*-timescale and accumulating the coactivities over the timescale of working or intermediate memory.

## Discussion

It has long been established that both *θ* and *γ* rhythms correlate strongly with the capacity for learning and memory, but the mechanisms by which they influence cognitive functions has remained unclear. These extracellular fields define the timescale of place cell coactivity, thereby controlling the “parcellation” of the information flow received by downstream networks. In particular, the synchronization of the processes taking place at the synaptic timescale, such as the processes controlled by the membrane time constant, the duration of receptor-mediated postsynaptic spike potentials, the rate of spike-timing dependent plasticity, and so forth, [71–73] is manifested at the network level as *γ*-frequency oscillations [74–78]. Processes that involve slower forms of synaptic plasticity, including slow-changing spiking thresholds [79–82], synchronize at the *θ*-frequency timescale. As a result, *θ*-oscillations provide lower-resolution temporal packaging of place cell coactivity [66, 83, 84], integrating spiking inputs from several cell assemblies over one or more *θ*-periods [85–87].

Our previous model, based on place cells that are independently *θ*-precessing, provided a self-consistent description of the hippocampal network’s function at the *θ*-timescale, which predicted an optimal integration window for reading out the information within the *θ*-range [30]. However, as the integration window became smaller, the spatial map encoded by independently precessing place cells failed to achieve correct spatial representation, which suggested to us the importance of additional synchronization at the *γ*-timescale. Here we developed a phenomenological model based on the assumption that the *γ*-rhythm not only controls the probability of the cell assembly spiking but also defines the temporal spread of the spikes produced by the cell assemblies around the troughs of the *γ*-wave. As a result, the model predicts that if the preferred *θ*-phases synchronize with the *γ*-troughs, then topological information about the given environment can be readily captured by integrating place cell coactivity at the *γ*-timescale. Thus, *γ*-synchronization of spiking activity is crucial for both encoding and reading out information from the cell assemblies arriving in “*γ*-packets” [61].

This result suggests a possible phenomenological explanation as to why reduction of the *γ*-wave amplitude correlates with impairments in learning, whether the cause is changes in the network’s synaptic physiology [88–91], psychoactive drugs [92–94], neurodegeneration, or aging [95, 96], whereas an increase of the *γ*-amplitude correlates with successful learning and retrieval of the learned information [97–101]. According to our model, reducing either the *γ*-amplitude or the diffused coupling between the *γ*-rhythm and place cell spiking activity, the latter being equivalent to lowering *β*, should increase learning times and lower the success rate in constructing topologically accurate cognitive maps. Vice versa, high *γ*-amplitude and strong coupling between the spike times and the *γ*-wave should result in more effective spatial learning.

## Methods

### Glossary

An *abstract simplex* of order *d*, *σ*^*d*^, is a set of (*d* + 1) elements, e.g., a set of (*d* + 1) active cells. Note that the subsets of the set *σ*^*d*^ form subsimplexes of *σ*^*d*^ and that a nonempty overlap of any two simplexes $\sigma_{1}^{d}$ and $\sigma_{2}^{d}$ is a subsimplex of both $\sigma_{1}^{d}$ and $\sigma_{2}^{d}$. A *simplicial complex* *Σ*_*σ*_ is a family of simplexes. The elements of a simplex *σ*^*d*^ can be visualized as vertices of *d*-dimensional polytopes: *σ*^0^ can be visualized as a point, *σ*^1^ as the ends of a line segment, *σ*^2^ as the vertices of a triangle, *σ*^3^ as the vertices of a tetrahedron, etc. [102]. A *clique* in a graph *G* is a set of fully interconnected vertices (i.e., a complete graph). Combinatorially, cliques have the same key properties as the abstract simplexes: any subcollection of vertices in a clique is fully interconnected, and hence forms a subclique. A nonempty overlap of two cliques $\varsigma_{1}^{d}$ and $\varsigma_{2}^{d}$ is a subclique in both $\varsigma_{1}^{d}$ and $\varsigma_{2}^{d}$. Therefore, cliques define abstract simplexes and thus the collection of cliques in a graph *G* defines a *clique simplicial complex* Σ_*ς*_(*G*).

### Choice of the simulated environment

In [30] we showed that the time required to learn a large spatial environment is approximately equal to sum of times required to learn its parts. Therefore, we simulated a non-preferential exploratory behavior in a small planar environment (1*m* × 1*m*) shown in Fig 1A, similar to those used in electrophysiological experiments [103].

The **Poisson spiking rate** of a place cell *c* at a point *r*(*t*) = (*x*(*t*), *y*(*t*)) is given by

$$\lambda_{c}\left(r\right)=f_{c}e^{-\frac{{\left(r-r_{c}\right)}^{2}}{2s_{c}^{2}}}$$

where *f*_*c*_ is the maximal firing rate and *s*_*c*_ defines the size of the place field centered at *r*_*c*_ = (*x*_*c*_, *y*_*c*_). The set of *s*_*c*_s and *f*_*c*_s in an ensemble of *N* place cells are lognormally distributed around a certain ensemble-mean firing rate *f* and a certain ensemble-mean place field size *s*, with the variances *σ*_*f*_ = *af* and *σ*_*s*_ = *bs*, respectively. Thus, a place cell ensemble is described by a triplet of parameters: (*s*, *f*, *N*) [29].

### *θ*-phase precession

As the rat moves over a distance *l*(*t*) into the place field of a cell *c*, the preferred spiking phase is

$$\varphi_{\theta ,c}\left(t\right)\approx 2\pi \left(1-l\left(t\right)/L_{c}\right),$$

where *L*_*c*_ ∼ 3*s*_*c*_ is the size of the place field [36, 104]. To simulate the coupling between the firing rate and the *θ*-phase, we modulated the original Gaussian firing rate by a *θ*-factor Λ_*θ*, *c*_(*φ*), giving

$$\Lambda_{\theta ,c}\left(\varphi \right)=e^{-\frac{{\left(\varphi -\varphi_{\theta ,c}\left(t\right)\right)}^{2}}{2\varepsilon_{c}^{2}}},$$

using the *θ*-component of the LFP recorded in wild type rats. The width *ε* of the Gaussian was defined in [30] to be the ratio of the mean distance that rat travels during one *θ*-cycle to the size of the place field, *ε* = 2*πv*/*Lω*_*θ*_, where *v* is the rat’s speed and *ω*_*θ*_/2*π* is the frequency of the *θ*-signal.

### *γ*-modulation

To incorporate the *γ*-rhythm into our model, we extracted the 30–80 Hz frequency band from the same LFP signal so that all the existing correlations between *θ* and *γ* waves are preserved, then we shifted the simulated place cell spiking times towards the troughs of *γ* amplitude by modulating their respective spiking rates with the additional Boltzmann factor [58],

$$\Lambda_{\gamma }\left(t\right)∼e^{-\beta_{\gamma }A_{\gamma }\left(t\right)},$$ (1)

where *A*_*γ*_(*t*) is the amplitude of the *γ*-wave and 1/*β*_*γ*_ is a formal parameter that plays the role of the effective temperature [105] (Fig 2). Simulating the net firing rate as a product of all three factors

$$\lambda_{net}=\lambda_{c}\left(x,y\right)\Lambda_{\theta ,c}\left(\varphi \right)\Lambda_{\gamma }\left(A_{\gamma }\right)$$

preserves spatial selectivity of spiking and the *θ*-precession (S8 Fig) and forces the preferred phases of the *θ*-phase precession *φ*_*c*_ into the *γ*-cycles, in accordance with the *θ*-*γ* theory [38, 41, 56].

### Temperature of the cell assemblies

In a vicinity of the *i*^*th*^ trough, the gamma signal has the form

$$A_{\gamma }\left(t\right)\approx A_{\gamma ,0}-A_{\gamma ,i}\cos\left(\omega_{i}t\right)\approx a_{\gamma ,i}+A_{\gamma ,i}\frac{\omega_{i}^{2}t^{2}}{2},$$ (2)

where the parameters *A*_*γ*,0_ are the mean amplitude of *A*_*γ*_; *A*_*γ*,*i*_ and *ω*_*i*_ are its instantaneous amplitude and frequency at the *i*^*th*^ trough *a*_*γ*,*i*_ = *A*_*γ*,0_ − *A*_*γ*,*i*_ and the index *i* runs over all troughs *i* ∈ *I*. Using the expansion Eq (2) in Eq (1) allows estimating the spread Δ_*i*_ of the spikes around the *i*^*th*^ through from the Gaussian variance as

$$\Delta_{i}^{2}=\frac{1}{\beta_{\gamma }A_{\gamma ,i}\omega_{i}^{2}}.$$ (3)

A priori, in order to accurately define the temporal spread of spikes produced by different cell assemblies at different times, the inverse effective temperature should be trough-specific, *β*_*γ*,*i*_. However, we consider a simplified case in which the average *β*_*γ*_ = 〈*β*_*γ*,*i*_〉_*i* ∈ *I*_ defines the coupling between the *γ*-wave and the place cell spike times across the entire hippocampal network.

The variance Eq (3) is about six times smaller than the instantaneous period, i.e.,

$$\frac{6}{\sqrt{\beta_{\gamma }A_{\gamma ,i}}\omega_{i}}\approx \frac{2\pi }{\omega_{i}},$$

which implies that the effective temperature is approximately equal to the mean *γ*-amplitude

$$\frac{1}{\beta_{\gamma }}\approx A_{\gamma },$$

where *A*_*γ*_ = 〈*A*_*γ*,*i*_〉_*i* ∈ *I*_. By normalizing *A*_*γ*_ with the standard deviation $\sigma_{\gamma }={\langle \sqrt{A_{\gamma }^{2}\left(t\right)-A_{\gamma ,0}^{2}}\rangle }_{t}$, *A* = *A*_*γ*_/*σ*_*γ*_, we obtain the scaled parameter *β* = *β*_*γ*_ *σ*_*γ*_, with the characteristic value

$$\beta =\frac{1}{A}.$$

### Cell types

The described approach can be applied to both the TroPyr and RisPyr cells. Mathematically, the “rising phases of *γ*” controlling the spiking of RisPyr cells correspond to the vicinities of peaks of the time derivatives of the *γ*-amplitude. Therefore, the spiking probability of RisPyr cells can be constrained by a factor similar to Eq (1), involving the derivative of the *γ*-amplitude, *A*′(*t*), which would override the *θ*-precession constraint (Λ_*θ*,*c*_(*φ*) = 1) in the vicinity of the *A*′(*t*)-peaks. The analysis of the mixed (RisPyr and TroPyr) ensembles is more complex and *sui generis*.

**Mathematical methods** required for this study are based on Persistent Homology Theory (see [29] and [106, 107]) implemented by the “JPlex” freeware package [108].

## Supporting Information

S1 FigAn illustration of the Alexandrov-Čech theorem.A: A spatial domain traversed by a short fragment of the simulated trajectory (black line). The locations where seven simulated place cells produced their spikes are marked by asterisks of seven different colors. B: The place fields (regions marked by ovals) cover the traversed space. The construction of the corresponding Čech complex N is illustrated on the following panels. C: Each element of the cover corresponds to a vertex of the Čech complex: vertices are shown by small colored discs. D: Every overlapping pair of place fields contributes a one-dimensional (1*D*) link to the Čech complex. The result is the 1*D* skeleton of N. E: Every triplet of place fields with a common intersection contributes a two-dimensional (2*D*) facet (triangle), which together form the 2*D* skeleton of the Čech complex. F: According the Alexandrov-Čech theorem, the 2*D* skeleton represents the topology of the cover shown on panel A, e.g., captures the central hole in the environment.(TIF)

S2 FigBrain rhythms modulate place cell spiking activity.A: Spike times precess with the *θ*-rhythm (red wave): as the rat traverses a place field, the corresponding place cell discharges at a progressively earlier phase in each new *θ*-cycle. These “preferred” phases of the *θ*-rhythm correspond to particular *γ*-cycles; the blue wave shows the net *θ* + *γ* amplitude. The synchronized spikes (shown by tickmarks colored according to the place fields traversed by the animal’s trajectory) cluster over the *γ*-troughs, yielding dynamical cell assemblies. B: Analogy between the stochastic particles (red dots) in a 1*D* potential (black curve) and the spread of spike times (tickmarks) around the *γ*-troughs (same black curve). If the temperature is high (dashed line, top panel), the particles spread diffusely over the potential landscape, and when the temperature is low (bottom panel), they are confined at the bottoms of the potential wells. A similar effect is produced if the place cell firing rate is modulated by the Boltzmann factor $e^{-\beta_{\gamma }A_{\gamma }(t)}$, where *A*_*γ*_(*t*) is the amplitude of the *γ*-wave and *β*_*γ*_ represents the inverse temperature. When *β*_*γ*_ is low, the spikes of the dynamical cell assemblies are “hot” (i.e., more spread in time), and when *β*_*γ*_ is large, the spikes are concentrated at the *γ*-troughs.(TIF)

S3 Fig Histograms of the *γ*-phases at the times of place cell spiking, as a function of the inverse effective temperature *β*. The cooler the cell assemblies, the more the spikes are coupled with the *γ*-troughs.
(TIF)

S4 FigFreezing out spurious loops.Timelines of the topological loops in the coactivity complex produced in the environment shown in Fig 1 for different integration windows (scale of *w*’s is shown on top) and for different effective temperatures 1/*β* (colorbar on the right). As the width of the integration window narrows, the number of spurious topological loops in the coactivity complex increases. For large *w*’s, spurious loops tend to disappear with learning (the times *T*_min_ when the correct topological structure of $T_{\sigma }$ emerges are marked by vertical dashed lines). For small *w*’s, some of these loops persist, indicating that the detected coactivity information is insufficient for eliminating spurious loops in $T_{\sigma }$. However, cooling down the coactivity complex suppresses the proliferation of the spurious loops: at *β* = 2 (bottom row) the coactivity complex has a correct structure at the integration window *w* ≈ (2/3)*T*_*θ*_.(TIF)

S5 Fig The effect of *γ*-synchronization on spatial learning. Each panel represents the results of simulating 150 neuronal ensembles at different effective temperatures 1/*β* (colorbar on the right) and different integration times *w* (scale shown above). Each dot represents a particular ensemble of *N*_*c*_ place cells with the mean place field size *s*. The maximal firing rates of the simulated neurons are distributed lognormally around *f* = 25 Hz (see Methods in [29, 30]). The color of the dot indicates the average time *T*_min_ required to encode an accurate map of the environment shown on Fig 2A, averaged over ten place field maps with the same (*s*, *N*). If the integration window is large (two left-most columns), *γ*-synchronization does not produce a strong effect on learning times. As the integration window becomes smaller, cooling the coactivity complex increases the scope of successful place cell ensembles. This implies that *γ*-synchronization increases the resilience of the hippocampal network in the face of variations of the place cell spiking parameters.
(TIF)

S6 Fig The effect of *γ*-synchronization on spatial learning. Each panel represents the results of simulating 150 neuronal ensembles at different effective temperatures 1/*β* (colorbar on the right) and different integration times *w* (scale shown above). Each dot represents a particular ensemble of *N*_*c*_ place cells with the mean place field size *s*. The maximal firing rates of the simulated neurons are lognormally distributed around *f* = 25 Hz (see Methods in [29, 30]). The color of the dot indicates the average time *T*_min_ required to encode an accurate map of the environment shown on Fig 2A, averaged over ten place field maps with the same (*s*, *N*). If the integration window is large (two left-most columns), *γ*-synchronization does not produce a strong effect on learning times. As the integration window becomes smaller, cooling the coactivity complex increases the scope of successful place cell ensembles. This implies that that *γ*-synchronization increases the resilience of the hippocampal network in the face of variations of the place-spiking parameters.
(TIF)

S7 Fig Freezing out the spurious loops in clique complexes. Timelines of the topological loops in the clique coactivity complex produced in the environment shown on Fig 1, for different integration windows (scale of *w*’s is shown on top) and different effective temperatures 1/*β* (colorbar on the right). The learning times *T*_min_ are marked by red vertical dashed lines. The qualitative dependence of the number of topological loops in the coactivity complex on the width of the integration window and the effective temperature 1/*β* are similar to the ones produced by the coactivity complex. However, the overall numbers of spurious topological loops is smaller, and the coactivity complex has a correct structure even at the smallest integration window *w* ≈ (2/5)*T*_*θ*_.
(TIF)

S8 Fig Simulated place fields and *θ*-precession are not affected by *γ* modulation. A: Place fields shown for *β* = 0, *β* = 1 and *β* = 2. B: The *θ*-phase/position diagram illustrating the *θ*-precession of a simulated place cell for *β* = 0, *β* = 1 and *β* = 2.
(TIF)
