# Supplementary figures and images for: Gamma Synchronization Influences Map Formation Time in a Topological Model of Spatial Learning
Source: PLoS Comput Biol. 2016 Sep 16;12(9):e1005114. doi: 10.1371/journal.pcbi.1005114 (PMC5026372; doi:10.1371/journal.pcbi.1005114)

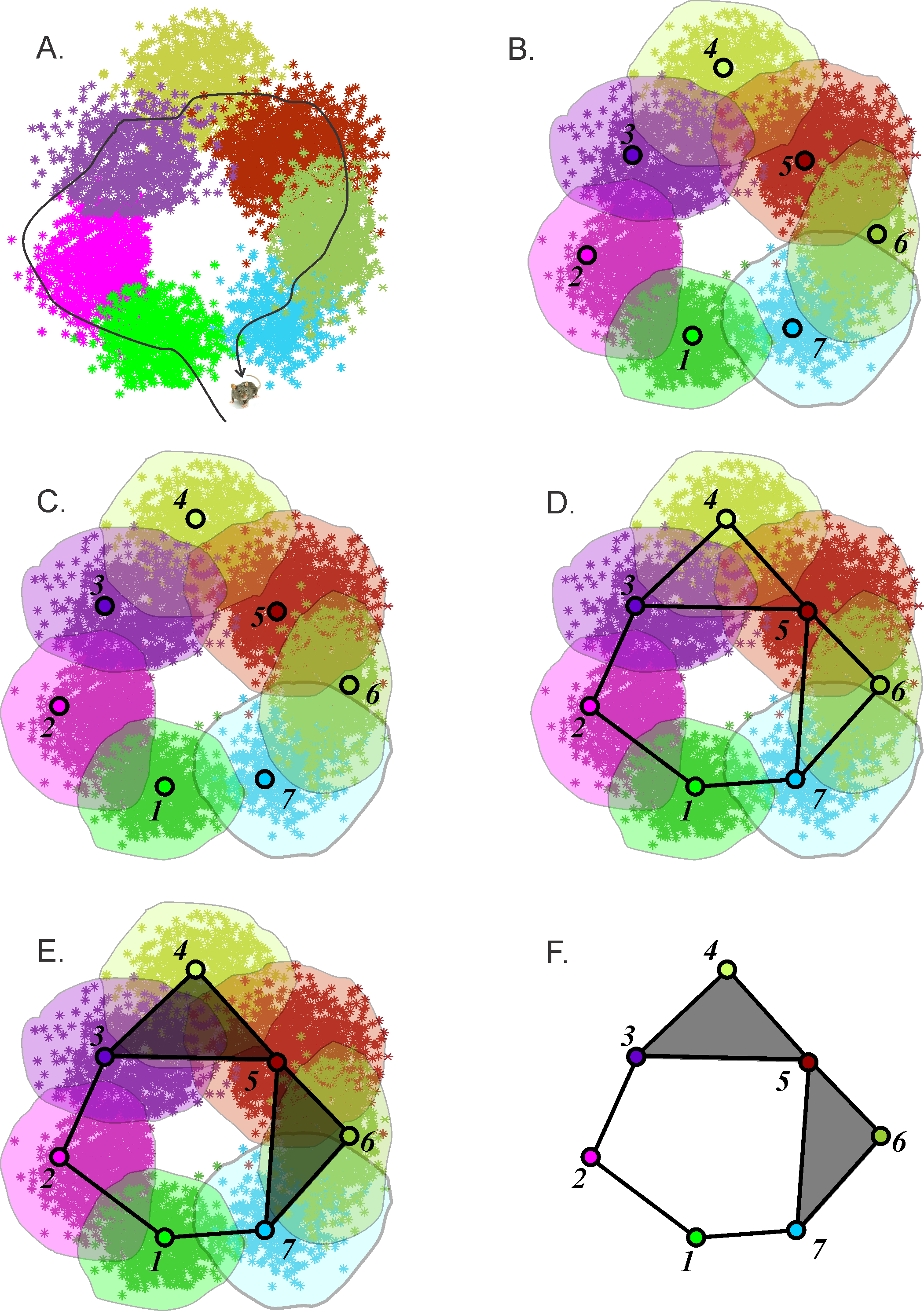

Supplement: S1 Fig — A: A spatial domain traversed by a short fragment of the simulated trajectory (black line). The locations where seven simulated place cells produced their spikes are marked by asterisks of seven different colors. B: The place fields (regions marked by ovals) cover the traversed space. The construction of the corresponding Čech complex N is illustrated on the following panels. C: Each element of the cover corresponds to a vertex of the Čech complex: vertices are shown by small colored discs. D: Every overlapping pair of place fields contributes a one-dimensional (1D) link to the Čech complex. The result is the 1D skeleton of N. E: Every triplet of place fields with a common intersection contributes a two-dimensional (2D) facet (triangle), which together form the 2D skeleton of the Čech complex. F: According the Alexandrov-Čech theorem, the 2D skeleton represents the topology of the cover shown on panel A, e.g., captures the central hole in the environment. (TIF) [file pcbi.1005114.s001.tif]

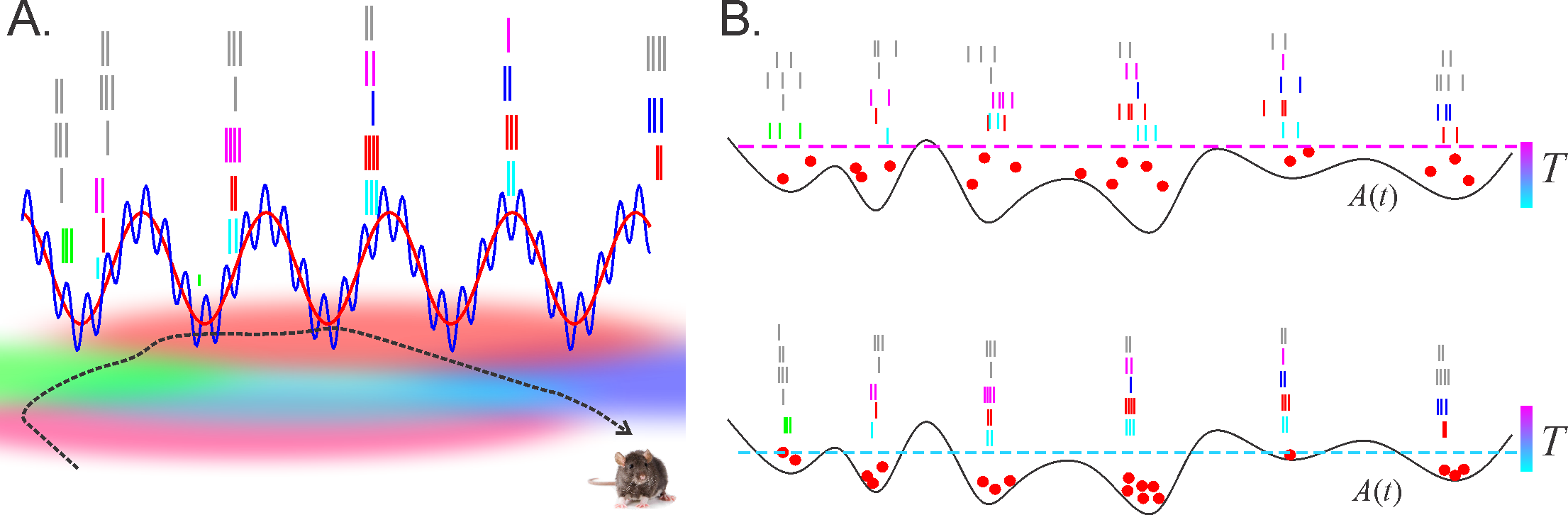

Supplement: S2 Fig — A: Spike times precess with the θ-rhythm (red wave): as the rat traverses a place field, the corresponding place cell discharges at a progressively earlier phase in each new θ-cycle. These “preferred” phases of the θ-rhythm correspond to particular γ-cycles; the blue wave shows the net θ + γ amplitude. The synchronized spikes (shown by tickmarks colored according to the place fields traversed by the animal’s trajectory) cluster over the γ-troughs, yielding dynamical cell assemblies. B: Analogy between the stochastic particles (red dots) in a 1D potential (black curve) and the spread of spike times (tickmarks) around the γ-troughs (same black curve). If the temperature is high (dashed line, top panel), the particles spread diffusely over the potential landscape, and when the temperature is low (bottom panel), they are confined at the bottoms of the potential wells. A similar effect is produced if the place cell firing rate is modulated by the Boltzmann factor e−βγAγ(t), where Aγ(t) is the amplitude of the γ-wave and βγ represents the inverse temperature. When βγ is low, the spikes of the dynamical cell assemblies are “hot” (i.e., more spread in time), and when βγ is large, the spikes are concentrated at the γ-troughs. (TIF) [file pcbi.1005114.s002.tif]

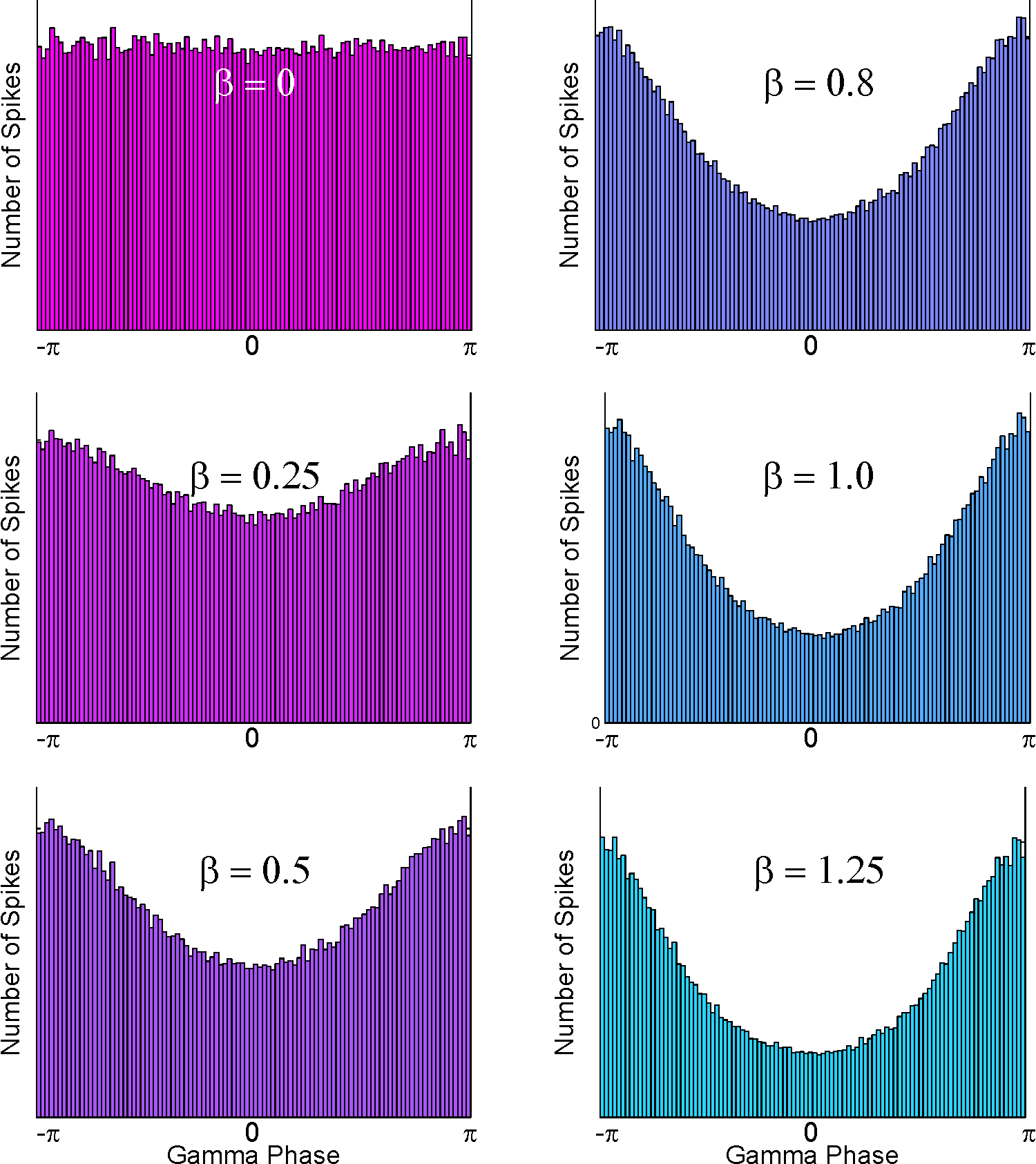

Supplement: S3 Fig — The cooler the cell assemblies, the more the spikes are coupled with the γ-troughs. (TIF) [file pcbi.1005114.s003.tif]

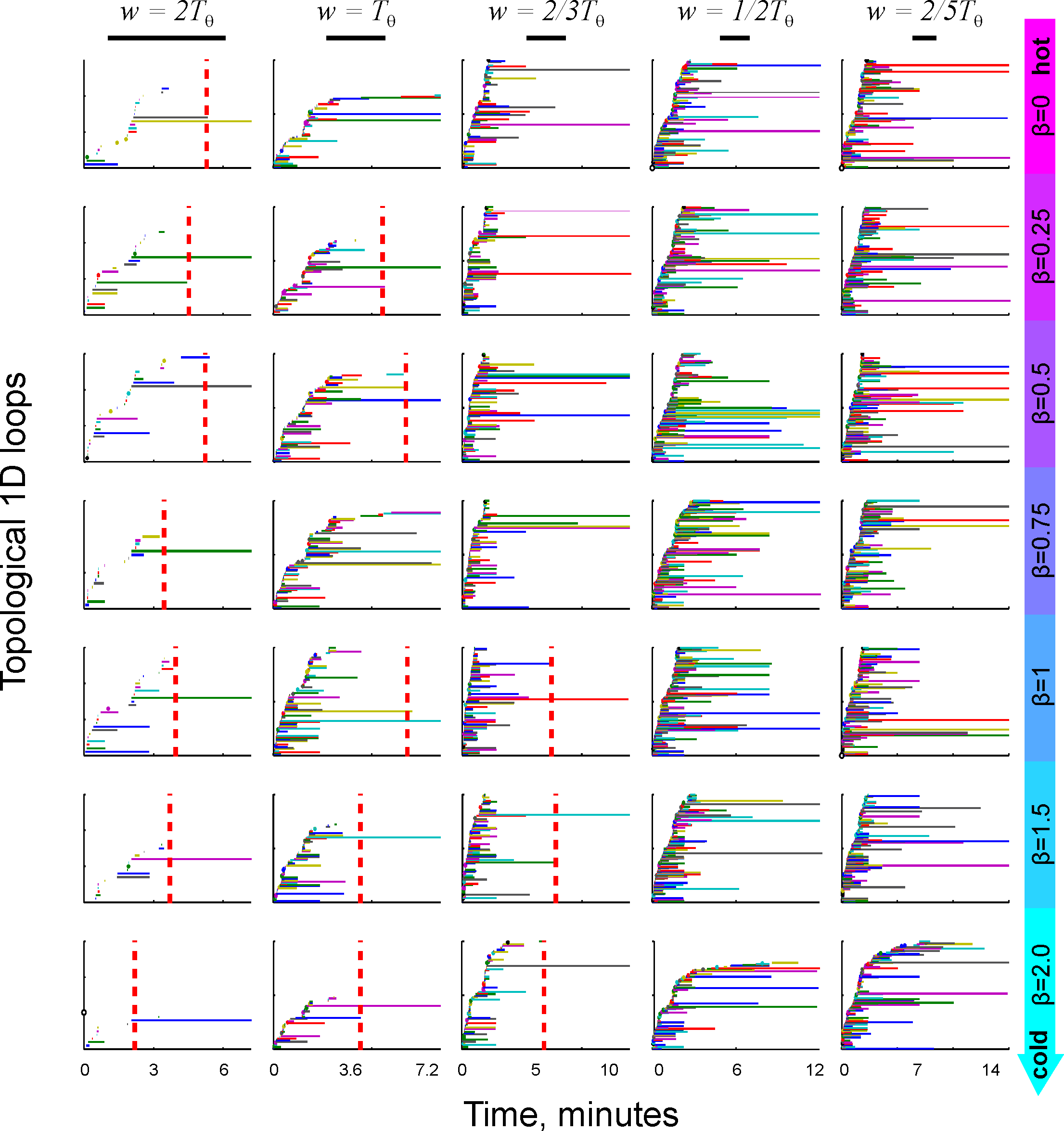

Supplement: S4 Fig — Timelines of the topological loops in the coactivity complex produced in the environment shown in Fig 1 for different integration windows (scale of w’s is shown on top) and for different effective temperatures 1/β (colorbar on the right). As the width of the integration window narrows, the number of spurious topological loops in the coactivity complex increases. For large w’s, spurious loops tend to disappear with learning (the times Tmin when the correct topological structure of Tσ emerges are marked by vertical dashed lines). For small w’s, some of these loops persist, indicating that the detected coactivity information is insufficient for eliminating spurious loops in Tσ. However, cooling down the coactivity complex suppresses the proliferation of the spurious loops: at β = 2 (bottom row) the coactivity complex has a correct structure at the integration window w ≈ (2/3)Tθ. (TIF) [file pcbi.1005114.s004.tif]

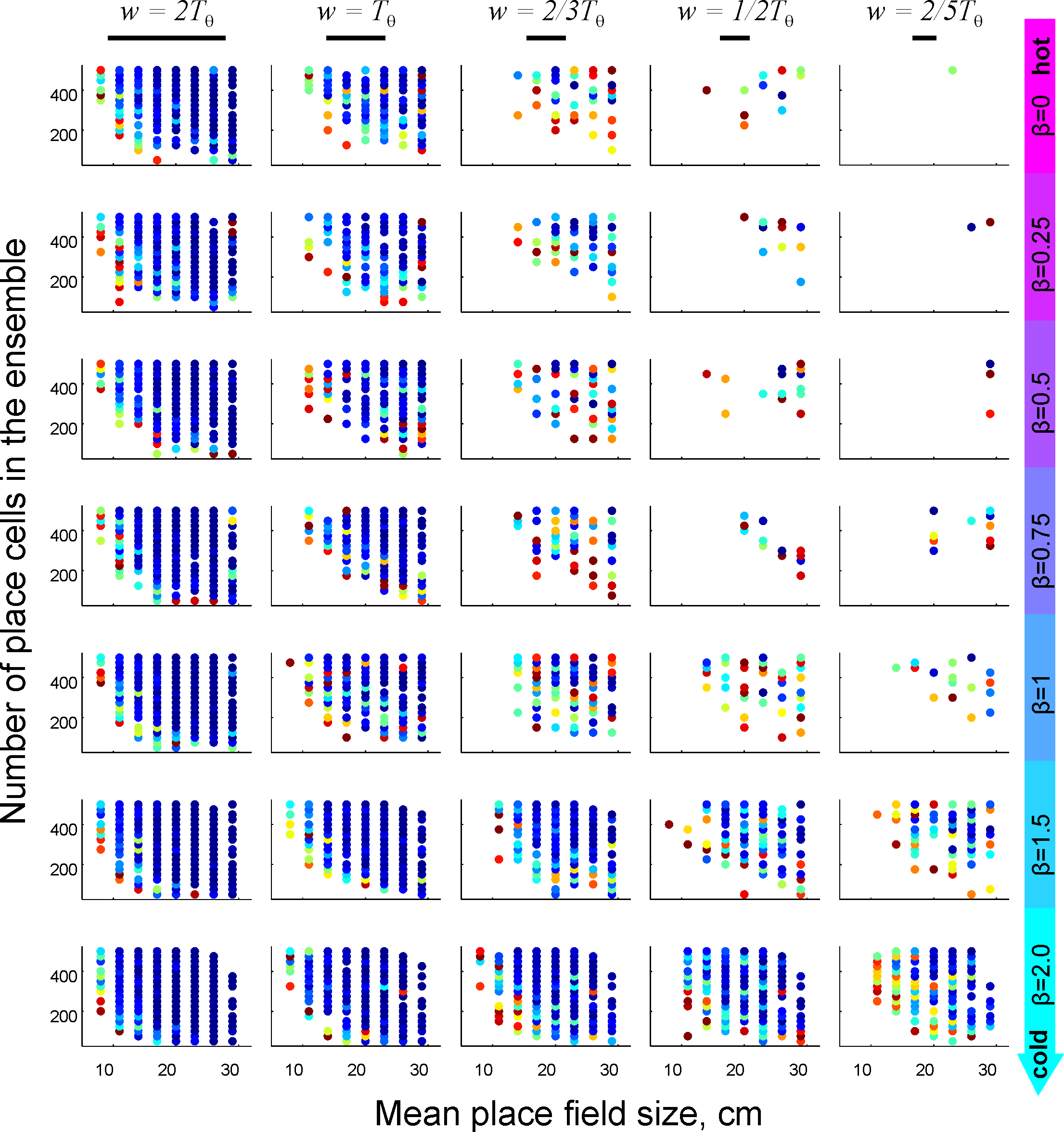

Supplement: S5 Fig — Each panel represents the results of simulating 150 neuronal ensembles at different effective temperatures 1/β (colorbar on the right) and different integration times w (scale shown above). Each dot represents a particular ensemble of Nc place cells with the mean place field size s. The maximal firing rates of the simulated neurons are distributed lognormally around f = 25 Hz (see Methods in [29, 30]). The color of the dot indicates the average time Tmin required to encode an accurate map of the environment shown on Fig 2A, averaged over ten place field maps with the same (s, N). If the integration window is large (two left-most columns), γ-synchronization does not produce a strong effect on learning times. As the integration window becomes smaller, cooling the coactivity complex increases the scope of successful place cell ensembles. This implies that γ-synchronization increases the resilience of the hippocampal network in the face of variations of the place cell spiking parameters. (TIF) [file pcbi.1005114.s005.tif]

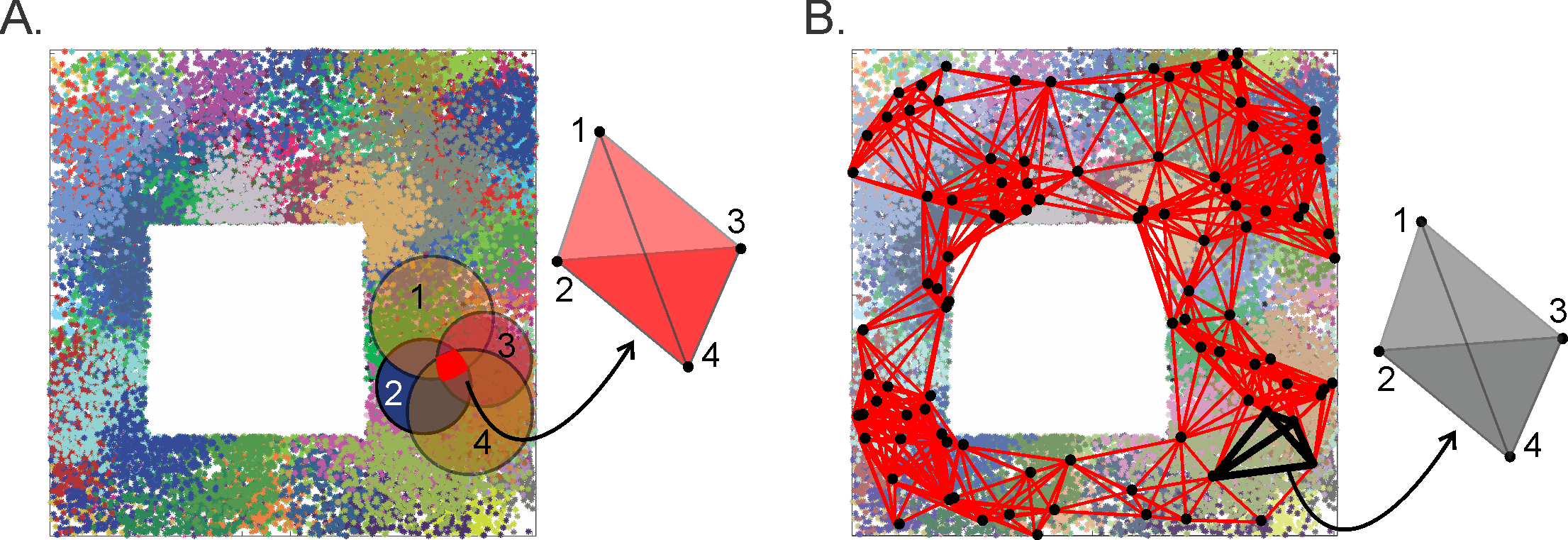

Supplement: S6 Fig — Each panel represents the results of simulating 150 neuronal ensembles at different effective temperatures 1/β (colorbar on the right) and different integration times w (scale shown above). Each dot represents a particular ensemble of Nc place cells with the mean place field size s. The maximal firing rates of the simulated neurons are lognormally distributed around f = 25 Hz (see Methods in [29, 30]). The color of the dot indicates the average time Tmin required to encode an accurate map of the environment shown on Fig 2A, averaged over ten place field maps with the same (s, N). If the integration window is large (two left-most columns), γ-synchronization does not produce a strong effect on learning times. As the integration window becomes smaller, cooling the coactivity complex increases the scope of successful place cell ensembles. This implies that that γ-synchronization increases the resilience of the hippocampal network in the face of variations of the place-spiking parameters. (TIF) [file pcbi.1005114.s006.tif]

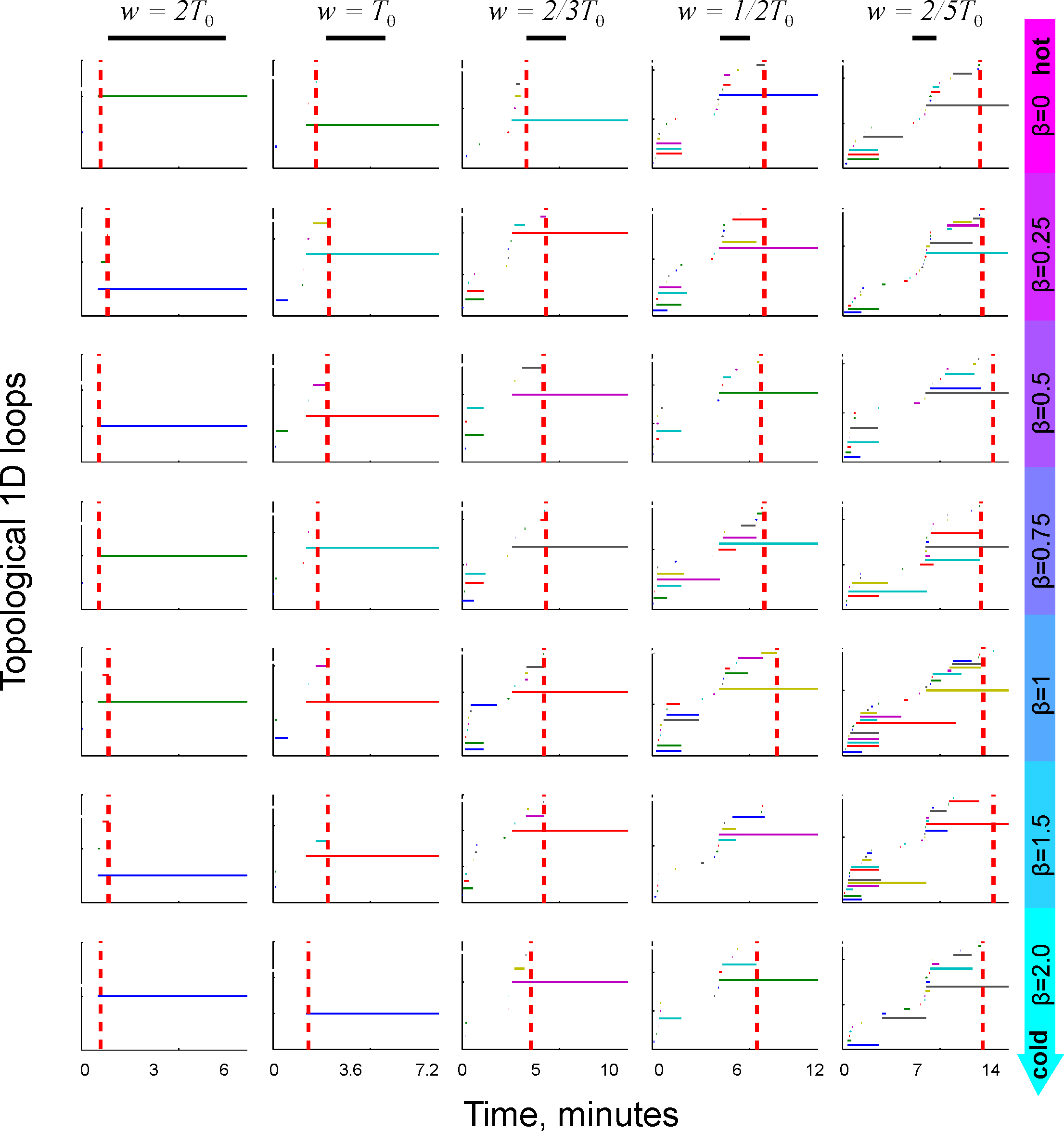

Supplement: S7 Fig — Timelines of the topological loops in the clique coactivity complex produced in the environment shown on Fig 1, for different integration windows (scale of w’s is shown on top) and different effective temperatures 1/β (colorbar on the right). The learning times Tmin are marked by red vertical dashed lines. The qualitative dependence of the number of topological loops in the coactivity complex on the width of the integration window and the effective temperature 1/β are similar to the ones produced by the coactivity complex. However, the overall numbers of spurious topological loops is smaller, and the coactivity complex has a correct structure even at the smallest integration window w ≈ (2/5)Tθ. (TIF) [file pcbi.1005114.s007.tif]

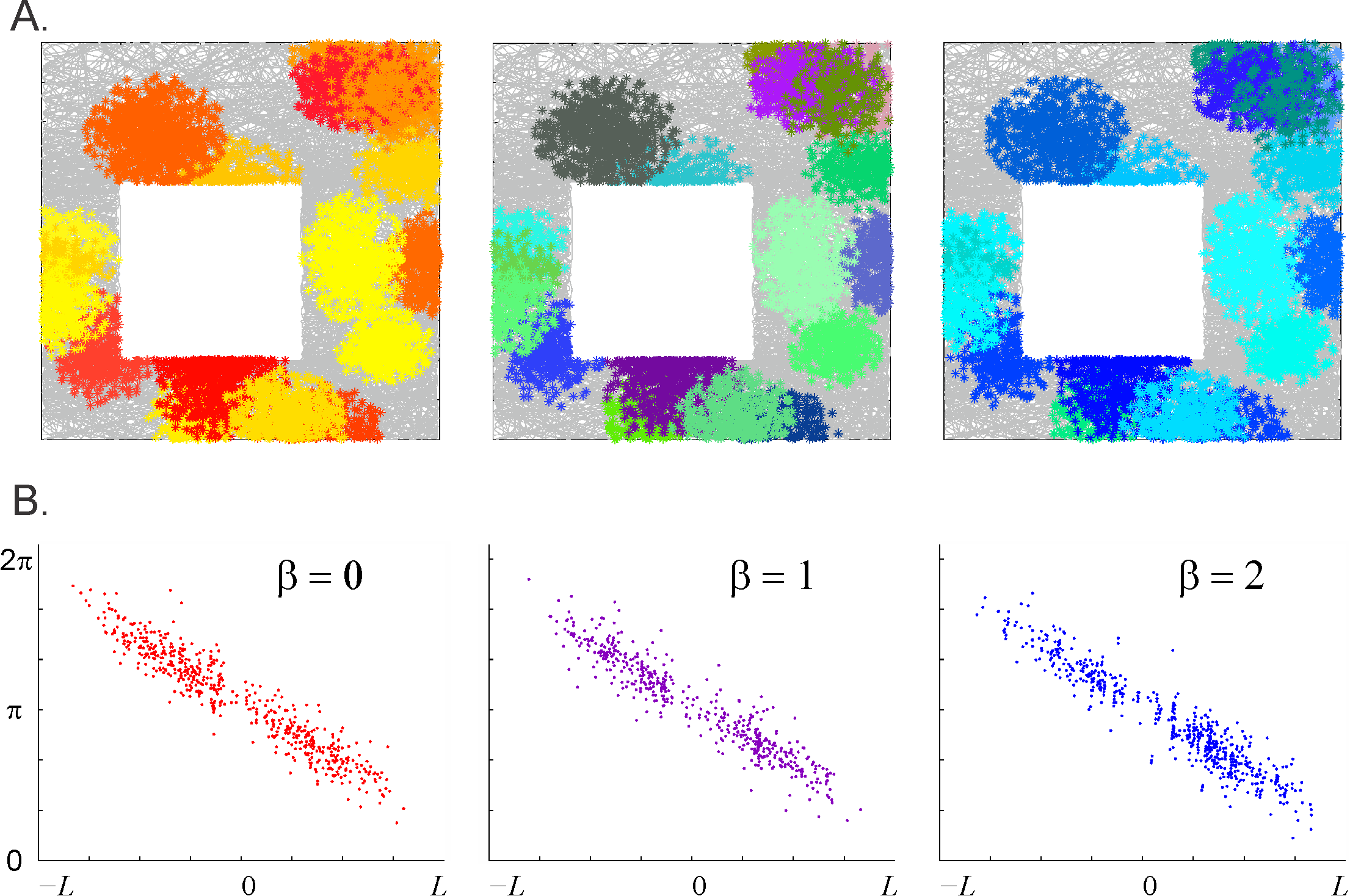

Supplement: S8 Fig — A: Place fields shown for β = 0, β = 1 and β = 2. B: The θ-phase/position diagram illustrating the θ-precession of a simulated place cell for β = 0, β = 1 and β = 2. (TIF) [file pcbi.1005114.s008.tif]
